# Atherosclerosis and Its Related Laboratory Biomarkers

**DOI:** 10.3390/ijms242115546

**Published:** 2023-10-24

**Authors:** Vittoriano Della Corte, Federica Todaro, Marco Cataldi, Antonino Tuttolomondo

**Affiliations:** Internal Medicine and Stroke Care Ward, Department of Health Promotion, Maternal and Infant Care, Internal Medicine and Medical Specialities (ProMISE) “G. D’Alessandro”, University of Palermo, Piazza delle Cliniche n.2, 90127 Palermo, Italybruno.tuttolomondo@unipa.it (A.T.)

**Keywords:** atherosclerosis, biomarkers, lipoproteins, microparticles, exosomes, miRNA, micro-RNA

## Abstract

Atherosclerosis constitutes a persistent inflammatory ailment, serving as the predominant underlying condition for coronary artery disease (CAD), peripheral artery disease (PAD), and cerebrovascular disease. The progressive buildup of plaques within the walls of medium- and large-caliber arteries characterizes the atherosclerotic process. This accumulation results in significant narrowing that impedes blood flow, leading to critical tissue oxygen deficiency. Spontaneous blockage of thrombotic vessels can precipitate stroke and myocardial infarction, which are complications representing the primary global causes of mortality. Present-day models for predicting cardiovascular risk incorporate conventional risk factors to gauge the likelihood of cardiovascular events over a ten-year span. In recent times, researchers have identified serum biomarkers associated with an elevated risk of atherosclerotic events. Many of these biomarkers, whether used individually or in combination, have been integrated into risk prediction models to assess whether their inclusion enhances predictive accuracy. In this review, we have conducted a comprehensive analysis of the most recently published literature concerning serum biomarkers associated with atherosclerosis. We have explored the potential utility of incorporating these markers in guiding clinical decisions.

## 1. Introduction

Atherosclerosis is a complex multifactorial disease and in industrialized and advanced countries is responsible for high cardiovascular disease (CVD) morbidity and death rates. Despite lifestyle management and pharmacotherapy improvements, it represents the primary cause of ischemic heart disease, stroke, and sudden death [1].

In the development of atherosclerosis disease, endothelial dysfunction plays a key role. The disturbance of the mechanisms responsible for regulating vascular homeostasis triggers an inflammatory reaction, which is regarded as the initial stage in the development of atheromatous plaques [2,3].

The primary hallmark of atherosclerosis is the chronic low-level inflammation occurring within the linings of large- and medium-sized arteries, linked to a lesion caused by the buildup of lipids beneath the endothelial layer. Possible causes of endothelial dysfunction include high levels of low-density lipoprotein (LDL), hypertension, diabetes mellitus, cigarette smoking, genetic alteration, a high level of plasma homocysteine concentrations, and infectious microorganisms such as herpesviruses or Chlamydia pneumoniae. In particular, atherogenesis is mainly caused by the accumulation of modified LDL [4]. The atherosclerotic lesion is characterized by a lipid core enclosed by a fibrous cap, which acts as a barrier between the core and the arterial lumen. One of the initial signs of atherosclerosis involves the deposition of lipids within the innermost layer of the arterial wall, known as the intima. Within the intima, lipids amass within the cells of the vascular wall, giving rise to the lipid core, and also in the extracellular space, where they bind with the components of the extracellular matrix. A pivotal moment in atherogenesis is the formation of “foam” cells. Foam cells have cytoplasm filled with lipid droplets [5].

Furthermore, endothelial dysfunction is also involved in subsequent steps of atherosclerosis by participating in plaque development and its rupture in atherosclerosis’s last efforts [3]. An increased endothelial dysfunction is considered an early indicator of atherogenesis [6]. In recent decades, various authors have focused on new potential biomarkers to identify atherosclerosis early.

## 2. Biomarkers of Atherosclerosis

In preceding years, serum biomarkers linked to atherosclerosis have been discovered, and they are correlated with an elevated risk of cardiovascular incidents such as atherosclerotic events. Several of these biomarkers, whether used individually or in conjunction, have been integrated into predictive models for assessing whether their inclusion enhances predictive accuracy. In this section we describe all the main biomarkers, from the oldest to the most recent.

### 2.1. Lipoproteins

Cholesterol deposition and chronic inflammation represent two main factors in the pathogenesis of atherosclerosis. The initial stage in the development of atherosclerosis involves the gathering of various plasma lipoproteins within the subendothelial space, particularly in areas where there is altered blood flow and endothelial dysfunction.

Plasma lipid concentrations exhibit a robust correlation with the susceptibility to cardiovascular diseases, and disorders in blood lipid profiles are acknowledged as risk factors for atherosclerosis, particularly in the advancement of plaque formation and thrombosis. Based on current research findings, the predominant source of lipids accumulated within the cells of arterial walls is attributed to low-density lipoproteins present in the bloodstream. Conversely, high-density lipoprotein (HDL) is regarded as protective because it facilitates the removal of lipids from cells, thereby impeding the development of foam cells [5].

Circulating lipoprotein particles have different physical and chemical parameters. Based on their size, density, and lipid and apolipoprotein composition, they can be separated into several classes. The primary source of lipid storage in atherosclerosis is represented by low-density lipoprotein, whereas high-density lipoprotein is not atherogenic. HDL has a recognized protective role, and its level is inversely correlated with atherosclerotic CVD risk [7].

Within the intima, reactive oxygen species (ROS) and reactive nitrogen species (RNS) bring about alterations in the functioning of lipids, carbohydrates, and proteins within cells. ROS, in particular, induce the oxidation of low-density lipoproteins (LDL) and facilitate the internalization of oxidized LDL (ox-LDL) by macrophages. Ox-LDL plays a pivotal role in the initiation and progression of atherosclerosis by inducing dysfunction in endothelial cells (ECs). This environment also leads to the expression of adhesion molecules for leukocytes and monocytes on the endothelial surface, including vascular cell adhesion molecule-1 (VCAM), intercellular adhesion molecule-1 (ICAM), E-selectin, and P-selectins. Consequently, these adhesion molecules facilitate the recruitment of monocytes, T lymphocytes, and mast cells into the intima of the vascular wall. Within the sub-endothelial space, molecules like interleukin-8 (IL-8), monocyte chemotactic protein-1 (MCP-1), and macrophage colony-stimulating factor (M-CSF) act to transform monocytes into macrophages [8].

**Figure 1:**
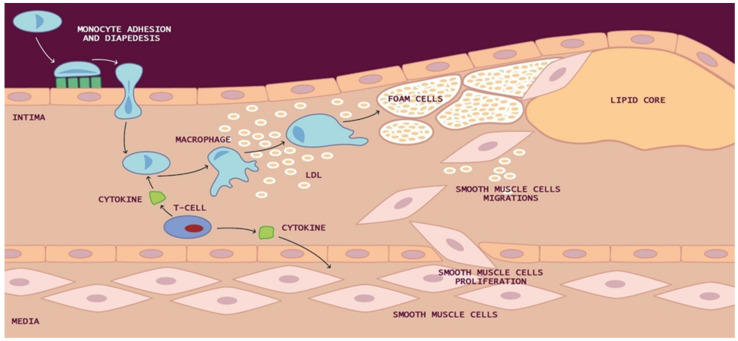
Development of atherosclerotic lesion: In the intima, reactive oxygen species (ROS) cause low-density lipoprotein (LDL) oxidation and promote the macrophages internalization of oxidized LDL (ox-LDL). Macrophages can also uptake LDL by micropinocytosis or in its aggregated form as cholesterol complexes by phagocytosis to form lipid-laden macrophages called “foam cells”. Yellow foam cells aggregate on the arterial walls and cause the development of fatty streaks. With the migration of smooth muscle cells (SMCs) from media to the intima and their proliferation, from the fatty streak is formed a fibrous atherosclerotic plaque cap.

Macrophages can also uptake LDL through micropinocytosis or in its aggregated state as cholesterol complexes through phagocytosis, leading to the formation of lipid-filled macrophages commonly referred to as “foam cells”. These yellowish foam cells congregate along arterial walls, initiating the formation of fatty streaks. As smooth muscle cells (SMCs) migrate from the media to the intima and proliferate, the fatty streak evolves into a fibrous atherosclerotic plaque cap (Figure 1).

The creation of intracellular cholesterol microcrystals triggers the activation of the inflammasome, which is a molecular complex composed of molecules that amplify the production of numerous pro-inflammatory cytokines and C-reactive protein (CRP). Significantly, the expansion of the plaque is linked to clinical complications and may increase the susceptibility to future thromboembolic events due to the presence of substantial cellular infiltrates and a thin fibrous cap [9].

**Figure 2:**
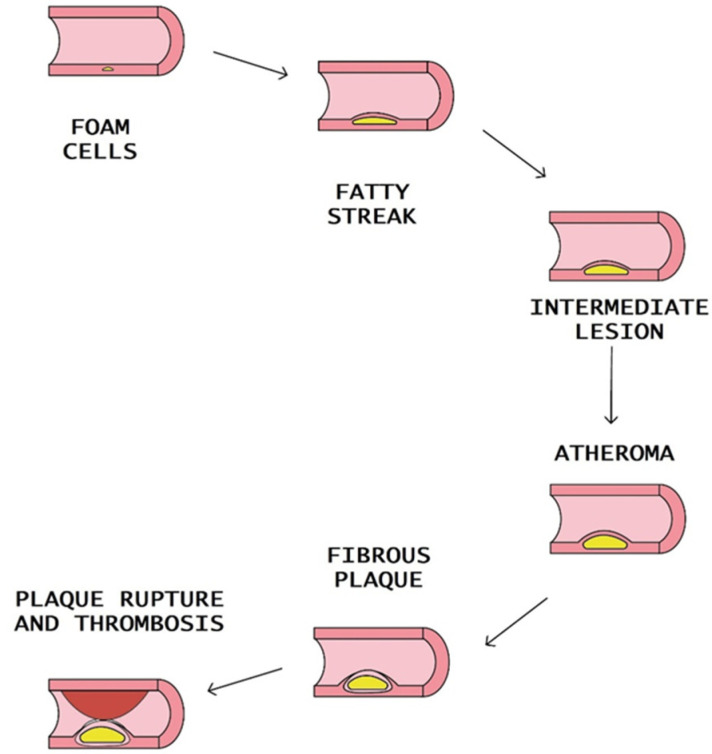
Steps of atheromatous plaque formation, progression, and rupture.

At advanced stages of atherosclerosis, the content of myeloid cells and lymphocytes in the plaque may predispose to the transformation into an “unstable plaque” with a thin fibrous cap. Macrophages and T lymphocytes secrete proteolytic enzymes such as metalloproteinase. The result is the reduction of the stability of the fibrous cap. The plaque rupture leads to a coagulation process, blood clot formation, thrombus formation, and blockade of the arteries [10] (Figure 2).

Various authors have conducted several studies to determine the association between the composition of circulating LDL and the risk of atherosclerosis and CVD. According to recent evidence, there are two main phenotypes of circulating LDL particles, A and B. The principal characteristic of phenotype A is the predominance of large buoyant LDL (lbLDL), and in phenotype B the principal characteristic is the predominance of small dense LDL (sdLDL) [11]. Phenotype B is correlated with various diseases, in particular with metabolic disorders like type 2 diabetes and obesity, and also represents a risk factor for coronary heart disease (CHD). This phenotype is related to plasma detection of elevated triglyceride (TG) levels, reduced HDL cholesterol (HDL-C), and high hepatic lipase activity [12]. A common finding in atherosclerosis patients’ serum is small dense LDL [13,14], and, according to the National Cholesterol Education Program (NCEPIII), the predominance of sdLDL is accepted as a risk factor for CVD [15]. Specific biochemical and biophysical properties of sdLDL are correlated with significant atherogenicity. Thanks to their small size, their penetration into the arterial wall is facilitated, so they represent a powerful source of cholesterol for developing atherosclerotic plaque. Longer circulation time results in multiple atherogenic modifications of sdLDL particles, increasing their atherogenicity.

An additional cardiovascular risk factor [16] is lipoprotein(a) (Lp(a)), which was first described in 1963. It contains another lipoprotein molecule covalently bound to apolipoprotein B, which has structural similarities to plasminogen. Lp(a) has pleiotropic effects on atherosclerotic cardiovascular disease (ASCVD), such as pro-inflammatory action on the arterial wall due to oxidized phospholipids, and atherogenic and pro-thrombotic activity due to the homology with plasminogen. Epidemiological, experimental, and genetic research suggests the likely existence of a causal connection between increased levels of Lp(a) and the risk of experiencing myocardial infarction (MI), stroke, and the development of calcific aortic valve stenosis [17].

Among the most common inherited metabolic disorders characterized by high levels of LDL cholesterol is familial hypercholesterolemia (FH). FH is a prevalent genetic disorder identified by substantially elevated LDL-C levels present from birth, leading to a considerably higher risk of atherosclerotic cardiovascular disease (ASCVD). Despite substantial progress in comprehending the condition and the availability of effective treatments, FH remains frequently undiagnosed and inadequately managed. Diagnostic scoring systems like the Dutch Lipid Clinic Network criteria, which take into account clinical manifestations, prove valuable in the identification of familial hypercholesterolemia (FH). Additionally, traditional risk factors and elevated lipoprotein(a) levels influence the progression of this condition [18].

The American Heart Association (AHA)/American College of Cardiology (ACC) Cholesterol guideline, published in 2018, and the 2019 ACC/AHA guideline on primary prevention of cardiovascular disease mention Lp(a) and high-sensitivity C-reactive protein (hsCRP) as risk enhancers [19]. High-sensitivity C-reactive protein (hsCRP) serves as an indicator of systemic inflammation and is upregulated due to vascular disease [20]. Research data indicate that besides its origin in the liver, hsCRP is also found within atherosclerotic plaques and damaged vessel walls, where it plays a role in its secretion, albeit in small quantities [20]. A recent analysis of the ACCELERATE trial (Assessment of Clinical Effects of Cholesteryl Ester Transfer Protein Inhibition with Evacetrapib in Patients at a High Risk for Vascular Outcomes) reported that Lp(a) was associated with major adverse cardiovascular events (MACE) only when hsCRP dosage reports levels ≥ 2 mg/L [21].

LDL particles within the human bloodstream can undergo various modifications, resulting in differences in their chemical composition. Consequently, the identification and measurement of these modified LDL particles have garnered considerable attention, as they may be associated with distinct risks related to atherosclerosis [11]. There has been significant advancement in the development of an inexpensive, rapid, and dependable method for quantitatively analyzing LDL subfractions. Statins and other lipid-lowering medications have shown positive effects in correcting the LDL profile. While numerous questions regarding the efficacy of reducing small dense LDL (sdLDL) in managing cardiovascular disease (CVD) risk remain unanswered, recent research suggests that the proportion of sdLDL-C may serve as a substantial marker for predicting CVD in various conditions associated with dyslipidemia [11] (Table 1).

### 2.2. Microparticles and Exosomes

Microparticles (MPs) and exosomes are the prominent extracellular vesicle (EVs) members. They are generated from different subcellular components. Both of them act as vehicles transporting bioactive molecules, including proteins, DNAs, mRNAs, non-coding RNAs, and lipids, between cells. Intercellular communication is an essential process in the pathogenesis of atherosclerosis. The regulation of atherosclerosis progression occurs with a substance exchange through exosomes among vascular smooth muscle cells, endothelial cells, macrophages, and other cell types [22]. MPs and exosomes deliver beneficial molecules or deleterious cargo to various tissues in normal physiologic and abnormal pathologic conditions. MPs are formed by cell membrane budding, while exosome release occurs after the fusion of multivesicular bodies (MVBs) with the plasma membrane into extracellular spaces through exocytosis [23]. Thanks to biological technologies like flow cytometry, electron microscopy, nanoparticle tracking analysis, and resistive pulse sensing, studies on microparticles and exosomes have grown rapidly in recent decades. MPs and exosomes, due to their roles in the pathogenesis of many diseases, are becoming new biomarkers and could be helpful in therapeutic applications of multiple conditions [24]. According to recent evidence, exosomes seem to be essential players in atherosclerosis [25].

Recently, many authors have focused on the relationship between exosomes and lipid disorders. Exosomes play a crucial role in lipid metabolism, mediating the process of lipid synthesis, transportation, and degradation, which are essential passages in atherosclerosis [25]. The detailed exosomal cargos involved in atherosclerosis remain largely unknown. Exosomes seem to be involved in lipid degradation and adipose tissue redistribution, suggesting that brown adipose tissue (BAT)-derived exosomes can reduce lipid accumulation and improve cardiac function [26]. Beyond the commonly studied micro-RNAs (miRNAs), some bioactive molecules could also be involved in the function of exosomes like long non-coding-RNAs (lncRNAs), circulating RNAs (circRNAs), and others. For example, the enzyme known as very long-chain acyl-CoA dehydrogenase (ACADVL), which is typically situated within the mitochondria, exhibited a significant abundance in exosomes originating from brown adipose tissue (BAT). These exosomes derived from BAT were found capable of transferring ACADVL as a biologically active protein into hepatic cells. The investigation of exosomal proteins and lipids in relation to atherosclerosis represents emerging research domains [27]. Thanks to their easy accessibility and their high degree of stability in body fluids, exosomes have emerged as rational biomarkers for various diseases. Recent research has shown that exosome-derived miRNAs can be easily isolated from different liquids. Theoretically, exosome-derived miRNAs are a better biomarker than circulating miRNAs in plasma/serum, while exosomes from specific cell types can be purified, increasing sensitivity and specificity [28,29]. Several miRNAs derived from exosomes have been studied. In particular, some of them (Mir-122-5p, Mir-27b-3p, Mir-101-3p, etc.) have been related to recurrent ischemic events in intracranial atherosclerotic disease [30]. Other exosomal miRNAs are associated with atherogenesis, such as Mir-92a-3p released by endothelial cells, Mir-30e, and Mir-92 [31].

Studies have shown that they are over-regulated in atherosclerosis and negatively related to plasma cholesterol and ATP-binding cassette transporter A1 (ABCA1) levels, representing a new biomarker for clinical diagnosis and treatment of coronary atherosclerosis [32].

Exosomal miRNAs that play a role in the development of atherosclerotic lesions include Mir-133a, Mir-155, Mir-21, Mir-210, Mir-126, and Mir-499. These discoveries have also positioned them as potential biomarkers for diagnosing, stratifying risk, and predicting prognosis [29,33].

Sorrentino et al. have reported a strong correlation between miRNAs encapsulated within circulating exosomes and the severity of atherosclerosis. Nevertheless, despite these encouraging findings, it is important to note that none of these biomarkers have undergone validation in large-scale cohort studies. Prior to the translation of exosomal biomarkers into clinical practice, it is imperative that they undergo thorough validation and receive accreditation [34]. Although several studies have revealed the potential of microparticles, exosomes, and exosomal miRNAs as new biomarkers, there are no standardized methods to isolate cell-specific exosomes and techniques to analyze exosomal contents with high sensitivity. Standardized isolation procedures are needed before integrating studies across different labs [35].

### 2.3. C-Reactive Protein

Inflammation has emerged as a promising target for therapy aimed at reducing the residual risk of atherosclerotic cardiovascular disease (ASCVD). Recent randomized controlled trials have shown that specific anti-inflammatory treatments can lead to improved cardiovascular outcomes [36]. Among the various inflammatory biomarkers, high-sensitivity C-reactive protein has received the most comprehensive validation in predicting ASCVD outcomes [37].

CRP, a non-specific indicator of inflammation, is a pentraxin produced by the liver in response to inflammation triggered by various mediators, including interleukin-1 (IL-1), interleukin-6 (IL-6), and tumor necrosis factor (TNF). The gene responsible for CRP is situated on the short arm of chromosome 1. At the transcriptional level, interleukin-6 is the principal regulator of the induction of CRP in hepatocytes, and this effect can be enhanced by interleukin-1β (IL-1β) [38].

The literature findings indicate that extrahepatic tissues, including various cell types within atherosclerotic plaques, can produce CRP. This cellular production of CRP may result in concentrations several times higher within atherosclerotic plaques compared with their levels in the bloodstream. Additionally, CRP receptors have been identified in various other cell types, such as neutrophils, resident macrophages, and endothelial cells.

CRP exercises pro-inflammatory and proatherogenic effects on different levels. In endothelial cells, CRP reduces nitric oxide and prostacyclin and increases endothelin-1, monocyte chemoattractant protein-1, interleukin-8, and cell adhesion molecules: it also increases plasminogen activator inhibitor-1 expression and activity.

There have been reports suggesting a potential causal role for CRP in the development of atherosclerosis. CRP has demonstrated the ability to upregulate cell adhesion molecules, enhance the release of MCP-1, facilitate the uptake of LDL into macrophages, and inhibit the production of Nitric oxide by destabilizing endothelial nitric oxide synthase (eNOS), all of which are critical features of atherosclerotic plaque formation. Furthermore, CRP also induces the polarization of monocytes towards the M1 phenotype and converts M2 monocytes into the M1 phenotype, thereby promoting the recruitment of monocytes into the plaque. Notably, circulating CRP binds to the cell membrane of activated monocytes but not to resting ones [39].

Several studies have shown that CRP independently predicts primary and secondary coronary heart disease events [40,41,42,43]; however, as CRP levels tend to rise in response to various inflammatory triggers, such as myocardial ischemia, these studies could not determine whether elevated CRP levels occurred before the onset of vascular disease. The role of CRP in predicting the risk of the first vascular event was initially demonstrated in the Physicians’ Health Study. This contentious matter was resolved through data from the prospective Physicians’ Health Study, which presented evidence showing that CRP levels measured using a “high sensitivity assay” had been elevated for decades prior to the first occurrence of acute ischemic events [44].

This study involved 22,000 healthy male physicians in the United States and found that men with baseline CRP values in the highest quartile had a threefold higher risk of acute coronary syndrome (MI; relative risk of 2.9) and a twofold higher risk of acute stroke (relative risk of 1.9) when compared with men in the lowest quartile of CRP values. Importantly, this risk association was independent of traditional risk factors [40].

Similarly, the Women’s Health Initiative observational cohort and the Nurses’ Health Study have shown that CRP can predict the risk of initial cardiovascular adverse events in women. Furthermore, CRP has been shown to reclassify the 10-year predicted risk of coronary heart disease (CHD) in approximately 30% of women [42,43]. However, other studies have raised questions about the additional predictive value of CRP. Danesh et al., in a prospective case-control study in Reykjavik, found that the predictive ability of CRP was only modest, with an odds ratio for CAD of 1.45 (95% CI, 1.25–1.68) [45].

In another study by Miller et al., data from the “Third National Health and Nutrition Examination Survey” were examined. They revealed that isolated elevation of CRP was rare, occurring in 4.4% of men and 10.3% of women, and it was often associated with abnormal or borderline risk factors. They also demonstrated that the risk of elevated CRP levels (>3 mg/L) could be attributed to any irregular or borderline risk factor in 78% of men and 67% of women [46].

According to data reported by “The Centers for Disease Control and Prevention” and the American Heart Association, CRP levels less than 1 mg/L, 1 to 3 mg/L, and greater than 3 mg/L are classified as low-, intermediate-, and high-risk values, respectively [47]. It has been suggested that CRP measurement may be particularly helpful in assessing the risk of intermediate-risk patients. Furthermore, several medications commonly used in the treatment of CHD, such as aspirin and statins, have been shown to reduce CRP levels [48] (Table 1).

### 2.4. Inflammatory Markers

Inflammation has been established as a pivotal factor in both the initiation and progression of the atherosclerotic process. In the early stages of atherosclerosis, the primary contributing factors include endothelial injury, abnormal lipid levels in the bloodstream, and hemodynamic stress. Flow-mediated inflammatory alterations in endothelial cells are believed to accompany the atherogenic process. Additionally, a hallmark of atherosclerosis is the buildup of lipids within macrophages, which readily triggers the activation of a multiprotein complex known as the inflammasome. [49,50,51,52].

Among the various inflammasomes, the NLR family pyrin domain-containing 3 (NLRP3) inflammasome stands out as the most extensively studied regulator implicated in the pathogenesis of cardiovascular diseases (CVDs). Human atherosclerotic lesions exhibit elevated expression of key components of NLRP3 [53] and inhibiting the NLRP3 inflammasome has been shown to notably reduce the progression of atherosclerosis.

Inflammasomes typically require two signals for assembly and activation [54]. Signal 1 primes the transcription of pro-IL-1β and NLRP3 inflammasome molecules via the nuclear factor kappa B (NF-κB) pathway. Signal 2, which can be triggered by the same stimuli, activates the inflammasome. This activation leads to the recruitment of the adapter molecule ASC (apoptosis-associated speck-like protein containing a caspase recruitment domain), which connects NLRP3 to pro-caspase-1. The formation of this complex then activates caspase-1, which catalyzes the cleavage of inactive pro-IL-1β into the major pro-inflammatory cytokine, IL-1β [55,56,57,58].

Additionally, other stimuli relevant to atherosclerosis, such as disturbed blood flow, can enhance the activation of the NLRP3 inflammasome [59,60].

Local production of IL-1β plays a crucial role in initiating the pro-inflammatory response, leading to the production of secondary inflammatory mediators like IL-6. IL-1β is a significant contributor to atherosclerosis and its complications, exerting multiple inflammatory effects on vascular endothelial cells, smooth muscle cells, and macrophages. For instance, IL-1β induces adhesion molecules in human endothelial cells and triggers autocrine production of platelet-derived growth factors, which can stimulate smooth muscle cell proliferation. Furthermore, this cytokine activates cells associated with innate immunity, particularly macrophages [61,62,63,64].

Another prominent player in atherosclerosis-related inflammation is IL-6. Interleukin-6 is a multifaceted cytokine that regulates both innate and adaptive immune systems, as well as the acute-phase response and chronic inflammation. Studies measuring IL-6 levels in blood samples of coronary heart disease patients have found elevated IL-6 levels in the coronary heart disease group compared with healthy individuals, with more severe disease correlating with higher levels of this inflammatory marker. Additionally, interleukin-6 levels significantly increase in cases of plaque rupture [65,66].

Signorelli et al. have highlighted how, among cytokines, IL-6 is the most extensively studied in the context of peripheral artery disease (PAD). Research has revealed that elevated serum levels of various markers associated with the acute inflammatory response, including IL-6, are higher in patients with type 2 diabetes. Consequently, individuals with type 2 diabetes have a twofold increased risk of developing PAD compared with those who do not have type 2 diabetes [67].

In patients with peripheral arterial disease (PAD), Signorelli et al. described how exercise affects inflammation levels. The concentration of muscle-derived IL-6 in the blood is directly related to the intensity of exercise and is also influenced by the type of exercise. Muscle-derived IL-6 exhibits significant pro-inflammatory properties and promotes the release of anti-inflammatory cytokines, such as IL1 receptor antagonist (IL-1-ra) and IL-10. Initially, the release of these substances into the bloodstream was thought to be a result of exercise-induced muscle damage. However, it should now be regarded primarily as a metabolic support mechanism for muscle metabolism during exercise. This support enhances the availability of glucose, promotes lipolysis, and facilitates the oxidation of fat [68,69].

Among the inflammatory markers studied in the context of atherosclerosis, Wachsmann-Maga et al. explored the role of leukotrienes (LTs), suggesting the potential for a pathophysiological link between atherosclerotic alterations and products derived from the metabolism of arachidonic acid [70]. Leukotrienes (LTs) are highly potent biologically active compounds that play a role in various cellular processes and function as mediators of inflammation. They belong to the eicosanoid group, which are signaling molecules involved in inflammation [70]. LTs are produced as part of the metabolic pathway that oxidizes essential fatty acids, such as arachidonic acid (AA) and eicosatetraenoic acid (EPA), through the action of the enzyme arachidonate 5-lipoxygenase. There are five main types of LTs: LTA4, LTB4, LTC4, LTD4, and LTE4. In individuals with stable and acute forms of coronary artery disease, there is an overproduction of various LTs. Notably, the levels of LTB4 and LTC4 in the blood are elevated in patients with cerebral ischemia. Similarly, patients with acute and chronic peripheral artery disease exhibit increased levels of LTE4 in their urine [70]. Furthermore, Maga P. et al. conducted a study that showed how successive measurements of urinary leukotriene E4 could be used to anticipate the lack of success in angioplasty, whether performed with or without stent implantation, for peripheral artery occlusive disease [71].

This suggests a clear trend associating cardiovascular atherosclerotic diseases with heightened LT production. However, the precise nature of this relationship remains unclear, and the impact of elevated leukotrienes on clinical manifestations of atherosclerotic diseases is still a subject of ongoing investigation [70].

### 2.5. Cells

Previous studies have demonstrated the significant role of T cells as key drivers and modulators in the pathogenesis of atherosclerosis. CD8^+^ T cells and CD4^+^ T cells are central in driving immune responses by recognizing peptides presented by major histocompatibility complex (MHC) class I on all nucleated cells and MHC class II on antigen-presenting cells (APCs), respectively. These responses occur in individuals expressing a specific MHC allele capable of binding the peptide epitope. The interaction between a specific T cell receptor (TCR) and co-stimulatory molecules provided by APCs activates T cells and leads to clonal proliferation. In experimental models of atherosclerosis, interactions between APCs and CD4^+^ T cells within the plaque lead to the secretion of various cytokines, many of which are pro-inflammatory [9,72,73,74,75,76].

Studies employing immunohistochemistry, single-cell RNA sequencing (scRNA-seq), and mass cytometry (CyTOF) have revealed that 25–38% of all leukocytes in human atherosclerotic plaques are CD3^+^ T cells. Research utilizing cellular indexing of transcriptomes and epitopes by sequencing (CITE-seq) has demonstrated that T cells constitute the majority of immune cells in human atherosclerotic plaques obtained from endarterectomy procedures [77,78,79,80,81,82].

Natural killer T (NKT) cells are categorized into two subgroups: invariant NKT (iNKT) cells (or type I), which possess limited variant TCRs, and type II NKT cells, which have more diverse T cell receptors (TCRs). Similar to CD8^+^ T cells, iNKT cells are capable of producing cytotoxic proteins such as perforin and granzyme B. Several studies conducted in Apoe^−/−^ mice and Ldlr^−/−^ mice fed either a Western or Chow diet have suggested that iNKT cells are pro-atherogenic. It is likely that iNKT cells promote atherosclerosis by releasing cytokines and activating other immune cells within the atherosclerotic lesion [83,84,85]. Under atherosclerotic conditions, macrophage-secreted inflammatory mediators are considered the predominant drivers of endothelial activation with subsequent monocyte adhesion and endothelial permeability. In the development of inflammation in atherosclerosis, various types of macrophages play crucial roles. These resident cell types typically remain relatively quiescent in healthy tissue and become activated in response to damage or antigenic stimuli, along with infiltrating monocytes/macrophages recruited due to pro-inflammatory signals. Macrophage activation can occur in response to both endogenous and exogenous triggers. For instance, macrophages can adopt a pro-inflammatory phenotype when exposed to microbial components like lipopolysaccharide, interferons (IFNs), toll-like receptor (TLR) engagement, or signaling by interleukin-4/interleukin-13 (IL-4/IL-13) [86,87]. Macrophage activation entails significant changes in gene expression profiles and the acquisition of cellular phenotypes specific to the activating stimulus.

M1-like macrophages primarily serve to eliminate pathogens and induce inflammatory responses by secreting pro-inflammatory mediators. These macrophages express receptors for IL-1, TLR, and co-stimulatory molecules, contributing to the initiation of the inflammatory response. M1-like macrophages produce pro-inflammatory cytokines such as IL-1, IL-6, TNF-α, interleukin-12 (IL-12), interleukin-23 (IL-23), and IL-13, as well as cytotoxic molecules like reactive oxygen species and nitrogen metabolites [88].

In atherosclerotic vascular diseases, macrophages play a pivotal role in plaque progression [89,90]. M1-like macrophages induce the recruitment and activation of other macrophages, T cells, B cells, and dendritic cells, thereby promoting inflammation and advancing atherosclerotic plaque development. The accumulation of intravascular lipids leads to the recruitment of monocytes to the atherosclerosis-prone areas, where they differentiate into macrophages. These macrophages subsequently undergo metabolic reprogramming in response to atherogenic stimuli present in the plaque microenvironment, such as modified lipoproteins and hypoxia. Upregulation of anabolic pathways like glycolysis and fatty acid synthesis, along with the pentose–phosphate pathway, which seem to facilitate atherogenesis, is a hallmark of activated immune myeloid cells within the developing atherosclerotic plaque [91].

Conversely, M2-like macrophages secrete anti-inflammatory and pro-fibrotic mediators and help limit inflammation, thus inhibiting the progression of atherosclerosis. Early regression of atherosclerosis can occur through increased apoptosis of cholesterol-laden macrophages, followed by the uptake of these apoptotic cells by neighboring macrophages [92] [Table 1].

### 2.6. Micro-RNA (miRNA)

Research into the pivotal roles of miRNAs in the development of atherosclerosis is an emerging field that has shown rapid growth in the number of miRNAs implicated in various aspects of atherosclerosis pathogenesis. These miRNAs are considered potential clinical biomarkers and are increasingly being explored as promising novel therapeutic targets to improve the management of atherosclerosis and cardiovascular diseases (CVDs) [93].

MiRNAs are a recently discovered class of highly conserved, single-stranded, non-coding endogenous RNAs approximately 22 nucleotides (nt) in length. They function by negatively regulating gene expression at the post-transcriptional level, either by inhibiting translation from messenger RNA (mRNA) or by promoting mRNA degradation [94]. MiRNAs play a crucial role in shaping the transcriptomes and proteomes of eukaryotic organisms. The human genome is estimated to contain at least 800 miRNAs [95], with many miRNA genes located within the introns of protein-coding genes, controlling approximately 30% of protein-coding genes. MiRNAs are recognized as potent regulators of various biological processes, including cell growth, proliferation, differentiation, migration, senescence, apoptosis, and angiogenesis [96,97]. Moreover, dysregulated miRNA expression and function are closely associated with various human pathologies, including cancer, diabetes, obesity, atherosclerosis, and CVDs [98,99,100].

The initial step in the development of atherosclerotic lesions involves endothelial dysfunction. E-selectin and vascular cell adhesion protein 1 (VCAM-1) increase vascular permeability and the expression of adhesion molecules, facilitating the migration of white blood cells into the vessel wall. miR-126, primarily expressed by endothelial cells, plays a significant role in this process. It inhibits the homing of white blood cells in the vessel wall by reducing the expression of VCAM-1 induced by tumor necrosis factor-alpha (TNF-α). Consequently, miR-126 reduces VCAM-1 expression in the endothelium, thus modulating detrimental vascular inflammation.

MiR-126 may exert paracrine effects on other vascular cells during atherosclerosis plaque development when transported within apoptotic bodies. MiR-155 and miR-221/222 exercised a role in the modulation of endothelial inflammation; in particular, they down-regulate the expression of NOsynthase (NOS), ETS-1 (E26 transformation-specific sequence) and its downstream inflammatory molecules in ECs [101,102].

In spontaneously hypertensive mice, it has been seen that levels of mir-125a/b are reduced. Consequently, there is an over-expression of endothelin 1, a potent vasoconstrictive peptide involved in vascular inflammation and atherosclerosis.

MiR-155 acts on the angiotensin II (AngII) pathway. AngII plays a crucial role in cardiac remodeling, atherosclerosis, and heart failure. MiR-155 may downregulate type 1 angiotensin-II receptor (AT1R) expression in both ECs and VSMCs in a post-transcriptional manner.

Following their adhesion to the endothelium, inflammatory cells, predominantly monocytes and T lymphocytes, migrate into the tunica intima. Within this intimal layer, these cells differentiate into macrophages and contribute to the inflammatory response. Several miRNAs play roles in promoting monocyte differentiation, and these include miR-155, miR-222, miR-424, and miR-503.

MiRNAs also play a regulatory role in both the differentiation and proliferation of vascular smooth muscle cells (VSMCs). The migration and proliferation of VSMCs are crucial factors in the progression of atherosclerosis [103].

Specifically, miR-21 has been identified as a promoter of vascular smooth muscle cell (VSMC) proliferation. On the other hand, miR-26a acts as a negative regulator of the transforming growth factor-alpha (TGF-α) signaling pathway, which induces VSMC proliferation and migration. Conversely, miR-145 and miR-133a inhibit proliferation and prevent VSMC phenotypic switching. MicroRNAs (miRNAs) play critical roles in the development and progression of atherosclerosis, a significant contributor to coronary artery diseases (CADs). Some miRNAs exhibit atherogenic effects, such as miR-122, miR-92a, and the miR-17-92 cluster, while others, like miR-30c, miR-148a, and miR-21, play atheroprotective roles. It is worth noting that several miRNAs can have conflicting roles in atherosclerosis; for example, miR-155 and miR-181b exhibit dual functions in various stages of the atherosclerosis process. Certain miRNAs, including miR-223, are considered hallmark molecules due to their significant impact on the initiation, progression, and plaque rupture phases of atherosclerosis.

A recent study by Sandeen Singh et al. showed that specific tissue-derived miRNAs are promising biomarkers for CAD disease, unstable CAD, and acute coronaric syndrome. In particular, tissue-derived miR-223-3 p and serum miR-122-5 p could reflect plaque instability. MiR-122-5 p also represents an increased risk of atherosclerosis in individuals with altered metabolic profiles and insulin resistance. Other miRNAs individuated were miR-146-3 p, miR-155-5 p, and miR-145-5 p; however, the data were less robust. MiR-233-3 p is a reliable biomarker in acute coronary syndrome [104].

Xin Wang et al., in the “China-Cardiovascular Disease Study”, evaluated the predictive ability of serum miRNAs for coronary artery disease. Among the 48 miRNAs analyzed, five miRNAs (miR-10a-5p, miR-126-3p, miR-210-3p, miR-423-3p, and miR-92a-3p) had better reliability and repeatability in serum, but the results show that only miR-423-3p had significant potential as a prognostic biomarker for CAD [105]. According to another study by Zhang et al., in patients with severe CAD requiring PCI, miR-32-3p, miR-3149, and miR-26a-5p have good diagnostic values for severe CAD [106].

A recent study conducted by Yao Lu et al. has shown that a specific miRNA expression profile, characterized by the up-regulation of miR-100, miR-133a/b, and miR-127, has the potential to identify atherosclerotic plaques with clinical evidence of vulnerability [103]. The existence of distinct miRNA profiles opens up the possibility that, in the future, specific microRNA signatures could be used as reliable and specific markers for early diagnosis and predictive characterization of acute atherosclerotic events. This represents a promising avenue for improving our ability to detect and manage atherosclerosis-related complications [98,101] (Table 2).

Regarding peripheral arterial disease (PAD), Signorelli et al. showed that miR-130a, miR-27b, and miR-210 could play a role in PAD since they are activated under hypoxic conditions. However, to date, any effective role of miRNAs as a target for PAD therapy remains to be clarified [107,108].

However, more studies on miRNAs are required to clarify their potential as a prognostic and diagnostic biomarker, to determine therapeutic opportunities and challenges, but also to overcome the limitations of miRNA application as therapeutic agents, including target specificity, safety, delivery, and efficiency [33,109,110].

## 3. Conclusions

Atherosclerosis is a chronic inflammatory disease that, in industrialized and advanced countries, is responsible for high cardiovascular disease morbidity and death rates. In recent decades, various authors have focused their attention on new potential biomarkers for the early identification of atherosclerosis.

Several studies have shown that CRP is an independent predictor of both primary and secondary coronary heart disease (CHD) events. Plasma lipid levels are strongly correlated with the risk of cardiovascular disease, and blood lipid disorder is recognized as a risk factor for atherosclerosis. A common finding in atherosclerosis patients’ serum is small dense LDL (sdLDL). An additional cardiovascular risk factor is lipoprotein(a) which has pleiotropic effects on ASCVD. MPs and exosomes are becoming new biomarkers and could be helpful in therapeutic applications of multiple diseases. According to recent evidence, exosomes are essential players in atherosclerosis for their crucial role in lipid metabolism. However, the detailed exosomal cargos involved in atherosclerosis remain largely unknown.

The research on the critical roles of miRNAs in atherosclerosis development is an emerging field. Still, more studies are needed to identify a precise miRNA panel to be used as a biomarker in atherosclerosis.

The recently published literature show that elevated serum levels of some biomarkers are associated with an increased risk of cardiovascular events. However, to date, no published studies demonstrate that specific treatment for those biomarkers leads to risk reduction, thus limiting the clinical utility of biomarker measurement in clinical practice.

The exception to this is the lipoproteins. The LDL and triglycerides lowering effect of statins and other lipid-lowering drugs were reported to have beneficial effects on cardiovascular risk.

The execution of further studies on the reduction of cardiovascular risk related to the modification of the most recent atherosclerosis markers that we have described is necessary to improve the clinical management of patients with atherosclerosis.

## Figures and Tables

**Table 1:** Laboratory biomarkers and their function in atherosclerosis.

Biomarkers	Function in Atherosclerosis
Ox-LDL	initiation and progression of atherosclerosis by inducing dysfunction in endothelial cells (ECs)
sdLDL	powerful source of cholesterol for developing atherosclerotic plaque
Lp(a)	pro-inflammatory action on the arterial wall due to oxidized phospholipids, and atherogenic and pro-thrombotic activity due to the homology with plasminogen
CRP	upregulate cell adhesion molecules, enhance the release of MCP-1, facilitate the uptake of LDL into macrophages
M1-like macrophages	induce the recruitment and activation of other macrophages, T cells, B cells, and dendritic cells, promoting inflammation and advancing atherosclerotic plaque development
M2-like macrophages	secrete anti-inflammatory and pro-fibrotic mediators and help limit inflammation, thus inhibiting the progression of atherosclerosis

**Table 2:** miRNAs function in atherosclerosis and their potential as a biomarker.

miRNAs	Function in Atherosclerosis and Their Potential as a Biomarker
Mir-122-5p, Mir-27b-3p, Mir-101-3p	related to ischemic events in intracranial atherosclerotic disease
Mir-30e, Mir-92, Mir-92a-3p miR-17-92	released by endothelial cells involved in atherogenesis
Mir-133a, Mir-155, Mir-21, Mir-210, Mir-126, and Mir-499	atherosclerotic lesion development
MiR-126	modulating in negative vascular inflammation
MiR-155 and MiR-221/222	down-regulate the expression of NOsynthase (NOS)
MiR-21	promoter of VSMC proliferation
MiR-26a	induces VSMC proliferation and migration
MiR-145 and MiR-133a	inhibit proliferation and prevent the phenotypic switch
MiR-30c, MiR-148a, and MiR-21	play atheroprotective role
MiR-223	driving initiation, progression, and plaque rupture steps
MiR-155, MiR-222, MiR-424, and MiR-503	promote monocyte differentiation
MiR-233-3 p, MiR-423-3p, MiR-32-3p, MiR-3149, and MiR-26a-5p	reliable as a biomarker in acute coronary syndrome
MiR-100, MiR-133a/b, and MiR-127	could identify plaques with clinical evidence of vulnerability

## Data Availability

No new data were created or analyzed in this study. Data sharing is not applicable to this article.

## Abbreviations

CAD	coronary artery disease
PAD	peripheral artery disease
CVD	cardiovascular disease
LDL	low-density lipoprotein
HDL	high-density lipoprotein
ROS	reactive oxygen species
RNS	reactive nitrogen species
ox-LDL	oxidized LDL
ECs	endothelial cells
VCAM-1	vascular cell adhesion molecule-1
ICAM-1	intercellular adhesion molecule-1
IL-8	interleukin-8
MCP-1	monocyte chemotactic protein-1
M-CSF	macrophage colony-stimulating factor
SMCs	smooth muscle cells
LbLDL	large buoyant LDL
SdLDL	small dense LDL
CHD	coronary heart disease
TG	triglyceride
HDL-C	HDL cholesterol
NCEPIII	National Cholesterol Education Program
Lp(a)	lipoprotein(a)
ASCVD	atherosclerotic cardiovascular disease
AHA	American Heart Association
ACC	American College of Cardiology
hsCRP	high-sensitivity C-reactive protein
MACE	major adverse cardiovascular events
MPs	microparticles
EVs	extracellular vesicles
MVBs	multivesicular bodies
BAT	brown adipose tissue
miRNAs	micro-RNAs
lncRNAs	long non-coding RNAs
circRNAs	circulating RNAs
ACADVL	very long-chain acyl-CoA dehydrogenase
ABCA1	ATP-binding cassette transporter A1
IL-1	interleukin-1
IL-6	interleukin-6
TNF	tumor necrosis factor
IL-1β	interleukin-1β
NLRP3	NLR family pyrin domain-containing 3
NF-κB	nuclear factor kappa B
MHC	major histocompatibility complex
APCs	antigen-presenting cells
TCR	T cell receptor
scRNA-seq	single-cell RNA sequencing
CyTOF	mass cytometry
CITE-seq	cellular indexing of transcriptomes and epitopes by sequencing
NKT	natural killer T
iNKT	invariant NKT
TCRs	T cell receptors
IFNs	interferons
TLR	toll-like receptor
IL-4/IL-13	interleukin-4/interleukin-13
IL-12	interleukin-12
IL-23	interleukin-23
NOS	NOsynthase
ETS-1	E26 transformation-specific sequence
